# Characterization of *MORE AXILLARY GROWTH* Genes in *Populus*

**DOI:** 10.1371/journal.pone.0102757

**Published:** 2014-07-18

**Authors:** Olaf Czarnecki, Jun Yang, Xiaoping Wang, Shucai Wang, Wellington Muchero, Gerald A. Tuskan, Jin-Gui Chen

**Affiliations:** 1 Biosciences Division, Oak Ridge National Laboratory, Oak Ridge, Tennessee, United States of America; 2 National Key Laboratory of Plant Molecular Genetics, Institute of Plant Physiology and Ecology, Shanghai Institutes for Biological Sciences, Chinese Academy of Sciences, Shanghai, China; 3 Key Laboratory of Molecular Epigenetics of MOE, Institute of Genetics and Cytology, Northeast Normal University, Changchun, China; Institute of Genetics and Developmental Biology, Chinese Academy of Sciences, China

## Abstract

**Background:**

Strigolactones are a new class of plant hormones that play a key role in regulating shoot branching. Studies of branching mutants in Arabidopsis, pea, rice and petunia have identified several key genes involved in strigolactone biosynthesis or signaling pathway. In the model plant Arabidopsis, *MORE AXILLARY GROWTH1* (*MAX1*), *MAX2*, *MAX3* and *MAX4* are four founding members of strigolactone pathway genes. However, little is known about the strigolactone pathway genes in the woody perennial plants.

**Methodology/Principal Finding:**

Here we report the identification of MAX homologues in the woody model plant *Populus trichocarpa*. We identified the sequence homologues for each MAX protein in *P. trichocarpa*. Gene expression analysis revealed that *Populus MAX* paralogous genes are differentially expressed across various tissues and organs. Furthermore, we showed that *Populus MAX* genes could complement or partially complement the shoot branching phenotypes of the corresponding Arabidopsis *max* mutants.

**Conclusion/Significance:**

This study provides genetic evidence that strigolactone pathway genes are likely conserved in the woody perennial plants and lays a foundation for further characterization of strigolactone pathway and its functions in the woody perennial plants.

## Introduction

Plant architecture plays a major role in determining photosynthetic light use efficiency and biomass yield. Optimal plant architecture is critical for achieving maximum carbon capture per unit land area especially when plants are grown in high-density stands. One recent breakthrough in the field of plant biology was the discovery of strigolactones (SLs) as a new class of plant hormones controlling shoot branching [1], [2]. More importantly, the synthesis of SLs in plants is regulated by the nutrient availability, particularly Pi deficiency, in soil and SLs exuded by roots serve as host recognition signals for symbiotic fungi [2]–[14]. Therefore, SLs are viewed as integrative signaling molecules that can couple nutrient availability and microbial symbiosis to the control of plant architecture and productivity. This breakthrough offers a new opportunity to dissect the interaction between plants and their environmental systems and to link this interaction to the control of plant architecture and productivity.

Substantial progress has been made in the last decade to understand the biosynthesis, signal transduction and physiological functions of SLs (reviewed by [12], [15]–[28]). *MORE AXILLARY GROWTH1* (*MAX1*), *MAX2*, *MAX3* and *MAX4* are four founding members of strigolactone pathway genes in the model plant Arabidopsis [29]–[32]. While *MAX1*, *MAX3* and *MAX4* are key genes involved in SL biosynthesis, *MAX2* is a key gene involved in SL signaling. *MAX1* encodes a cytochrome P450 monooxygenase [30]. *MAX3* and *MAX4* encode two carotenoid cleavage dioxygenases (CCD), CCD7 and CCD8, respectively [29], [31]. *MAX2* encodes an F-box leucine-rich protein [32]. Loss-of-function mutations in each of these four *MAX* genes resulted in increased shoot branching [29]–[32]. Orthologues for *MAX* genes have been identified and corresponding mutants have been characterized in pea (*ramosus, rms*), rice (*dwarf, d; high tillering dwarf, htd*) and petunia (*decreased apical dominance, dad*) (reviewed by [15]–[17], [19]–[21], [23]). In addition to *MAX* genes, several other genes involving in SL biosynthesis or signaling have been identified. For example, *D27* encoding a novel iron-containing protein is involved in SL biosynthesis [33], [34]. *PLEIOTROPIC DRUG RESISTANCE1* (*PDR1*) encoding an ATP-binding cassette transporter functions as a cellular SL exporter [35]. *D14* encoding a protein of the α/β-fold hydrolase superfamily is involved in SL signaling and likely functions as a receptor for SLs [36]–[40]. D53, a member of class I Clp ATPase protein family, is a substrate of the SCF^D3^ ubiquitination complex and the degradation of D53 protein is promoted by SL and is dependent on D14 and D3 (a rice ortholog of MAX2) [41], [42]. The degradation of BES1, a positive regulator in brassinosteroid signaling pathway, is also dependent on MAX2 [43].

However, despite this important discovery, little is known about this new class of plant hormones in perennial woody plants. So far, among perennial woody plants, strigolactone pathway genes have only been recently studied in willow [44], [45]. Because differences have been observed between monocots and dicots, between herbaceous and woody plants, and between annual and perennial plants, it has remained elusive whether the SL pathways are conserved in the perennial woody plants, such as *Populus*. For example, studies in herbaceous plants (such as Arabidopsis and pea) suggested that SLs act as long-distance signaling molecules that can be transported from roots to shoots to exert functional control on shoot branching. However, the woody perennial plants, such as *Populus*, are typically several orders of magnitude taller than annual herbaceous plants. This raises the question of whether SL can function similarly in *Populus*.

As a first step towards exploring SL pathways in the model woody plant *Populus,* we conducted a genome-wide search of sequence homologues of strigolactone pathway genes in the sequenced genome of *Populus trichocarpa* [46]. By using Arabidopsis strigolactone pathway genes as templates, we identified *Populus* sequence homologues for each of those four founding members of strigolactone pathway genes, namely *MAX1*, *MAX2*, *MAX3* and *MAX4*. We found that *Populus MAX* paralogous genes are differentially expressed across various tissues and organs. We showed that *Populus MAX* genes could complement or partially complement the shoot branching phenotypes of corresponding Arabidopsis *max* mutants. These findings provide genetic evidence that SL pathways are likely conserved in the woody perennial plants.

## Materials and Methods

### Plant Materials and Growth Conditions

Arabidopsis wild type Columbia-0 (Col-0) and mutant *max1-4* (SAIL_25_A05, ABRC stock #: CS862413) and *max2-4* (SALK_028336) were obtained from the Arabidopsis Biological Resources Center (Columbus, Ohio). Arabidopsis *max3-12* mutant has been previously described [47]. Seeds were surface sterilized by serial washing with 96% (v/v) ethanol, 20% (v/v) household bleach supplemented with 0.05% (v/v) Tween-20, and water, and placed at 4°C for 2 days. Seeds were subsequently plated on ½ Murashige and Skoog (MS) medium [48] supplemented with 1% (w/v) sucrose and 0.8% (w/v) agar, and germinated in 12 h/12 h photoperiod at 23°C, approximately 90 µmol photons m^−2 ^s^−1^. Seven day-old Arabidopsis seedlings were transferred from ½ MS medium to soil and grown in growth chambers at 23°C, approximately 125 µmol photons m^−2 ^s^−1^ with 14 h/10 h (long-day conditions) or 10 h/14 h (short-day conditions) photoperiods.

### Cloning of *Populus MAX* Homologous Genes

The full-length open reading frame (ORF) of each *PtrMAX* gene was determined according to the sequence information available at Phytozome [46], [49]. Gene-specific primers were designed to amplify the full-length ORF of each *PtrMAX* gene from cDNA library derived from RNA isolated from leaves and roots of *Populus trichocarpa* plants. Subsequently, the full-length ORF of each *PtrMAX* gene was introduced into the pENTR vector by using pENTR/D-TOPO Cloning Kit (Life Technologies). The cloned RT-PCR products were validated by sequencing and then transferred into plant Gateway destination vector pGWB502Ω (2×CaMV35SΩ) [50] using LR clonase (Life Technologies). All primers used for cloning are listed in Table S1.

### Genetic Complementation

pGWB502Ω (2×CaMV35SΩ) binary vectors containing *35S:PtrMAX* plasmid were transformed into Arabidopsis *max* mutants via *Agrobacterium tumefaciens* strain GV3101 mediated flower dipping transformation [51], [52]. T1 transformants were selected using 20 µg/L hygromycin B. A minimum of 20 independent transgenic lines were selected for each transgene. Two independent transgenic lines were used for further studies.

When plants reached maturity, the number of primary rosette-leaf branches was counted. A minimum of 10 individual plants per genotype were used.

### RT-PCR

To examine the absence or presence of Arabidopsis *MAX* transcript in the *max* mutant, total RNA was extracted from 7-day-old seedlings using the Invisorb Spin Plant Mini Kit (Stratec Molecular). Two µg of total RNA were reversely transcribed to cDNA using Fermentas RevertAid Reverse Transcriptase (Thermo Scientific). For semi-quantitative RT-PCR, Arabidopsis *MAX*-specific primers spanning the full-length ORF of *MAX* gene were used. PCR amplification of *AtACTIN8 (AtACT8)* served as a control.

For the examination of expression of *Populus MAX* genes in Arabidopsis transgenic lines, *PtrMAX* gene-specific primers were used. All primers used for semi-quantitative RT-PCR are listed in Table S1.

### Quantitative RT-PCR (qRT)

qRT was conducted to examine the transcript level of each *PtrMAX* genes across various tissues and organs using a StepOnePlus (Applied Biosystems), Maxima SYBR Green/ROX qPCR Master Mix (Thermo Scientific) and cDNA corresponding to 80 ng RNA in a total volume of 25 µl. Three biological replicates were used for qRT analysis. The following cycling conditions were used for PCR: 10 min at 95°C, 35 cycles of 15 s at 95°C and 60 s at 60°C. The transcript level was normalized against that of *PtrACTIN5 (PtrACT5)*. Gene-specific qRT primers were designed using QuantPrime [53]. All primers used for qRT analysis are listed in Table S1.

## Results

### Sequence Homologues of MAX Proteins in *Populus*

*MAX1*, *MAX2*, *MAX3* and *MAX4* are four founding members of SL pathway genes in the model plant Arabidopsis. In order to identify sequence homologs of *MAX* genes in *Populus trichocarpa* (hereafter referred to as *Populus*), we used amino acid sequences of Arabidopsis MAX proteins as templates to perform protein homology search in the fully-sequenced *Populus* genome.

**Figure 1:**
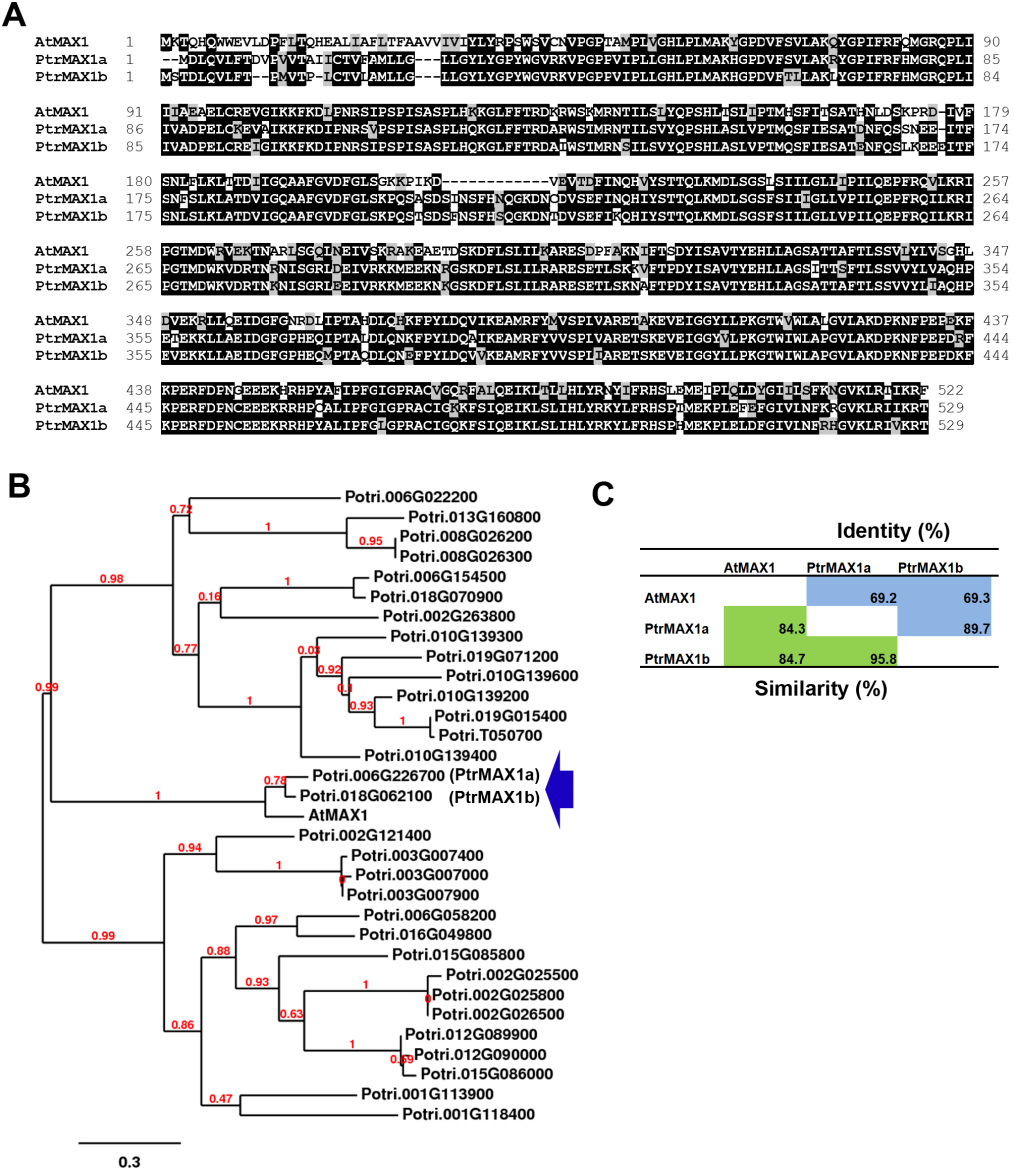
Bioinformatic analysis of MAX1 proteins from *Populus* and *Arabidopsis*. (**A**) Amino acid sequence alignment. (**B**) Phylogenetic analysis of *Populus* sequence homologues of *Arabidopsis* MAX1 protein. (**C**) Amino acid sequence similarity and identity.

Based on the current annotation by TAIR (http://www.arabidopsis.org) [54] and Phytozome (www.phytozome.net) [49], Arabidopsis *MAX1* (*AtMAX1*) has two transcript variants. The primary variant encoded by gene locus At2g26170.1 was used as a template for searching protein sequence homologues encoded by the *Populus* genome using the “Protein Homologs” search tool at Phytozome. The search identified two close sequence homologues encoded by loci Potri.006G226700 and Potri.018G062100, designated as PtrMAX1a and PtrMAX1b, respectively (Figure 1). Based on the current annotation by Phytozome, *PtrMAX1a* has three transcript variants and *PtrMAX1b* has four transcript variants. The primary variants (e.g., Potri.006G226700.1 and Potri.018G062100.1) were used for subsequent analysis. PtrMAX1a showed approximately 84% similarity and 69% identity with AtMAX1 at the amino acid level. PtrMAX1b showed approximately 85% similarity and 69% identity with AtMAX1 at the amino acid level. PtrMAX1a and PtrMAX1b each other shared approximately 96% similarity and 90% identity at the amino acid level. The number of amino acids of PtrMAX1a (529 aa) and PtrMAX1b (529 aa) are comparable to that of AtMAX1 (522 aa). No other *Populus* proteins showed more than 43% similarity with AtMAX1 at the amino acid level.

**Figure 2:**
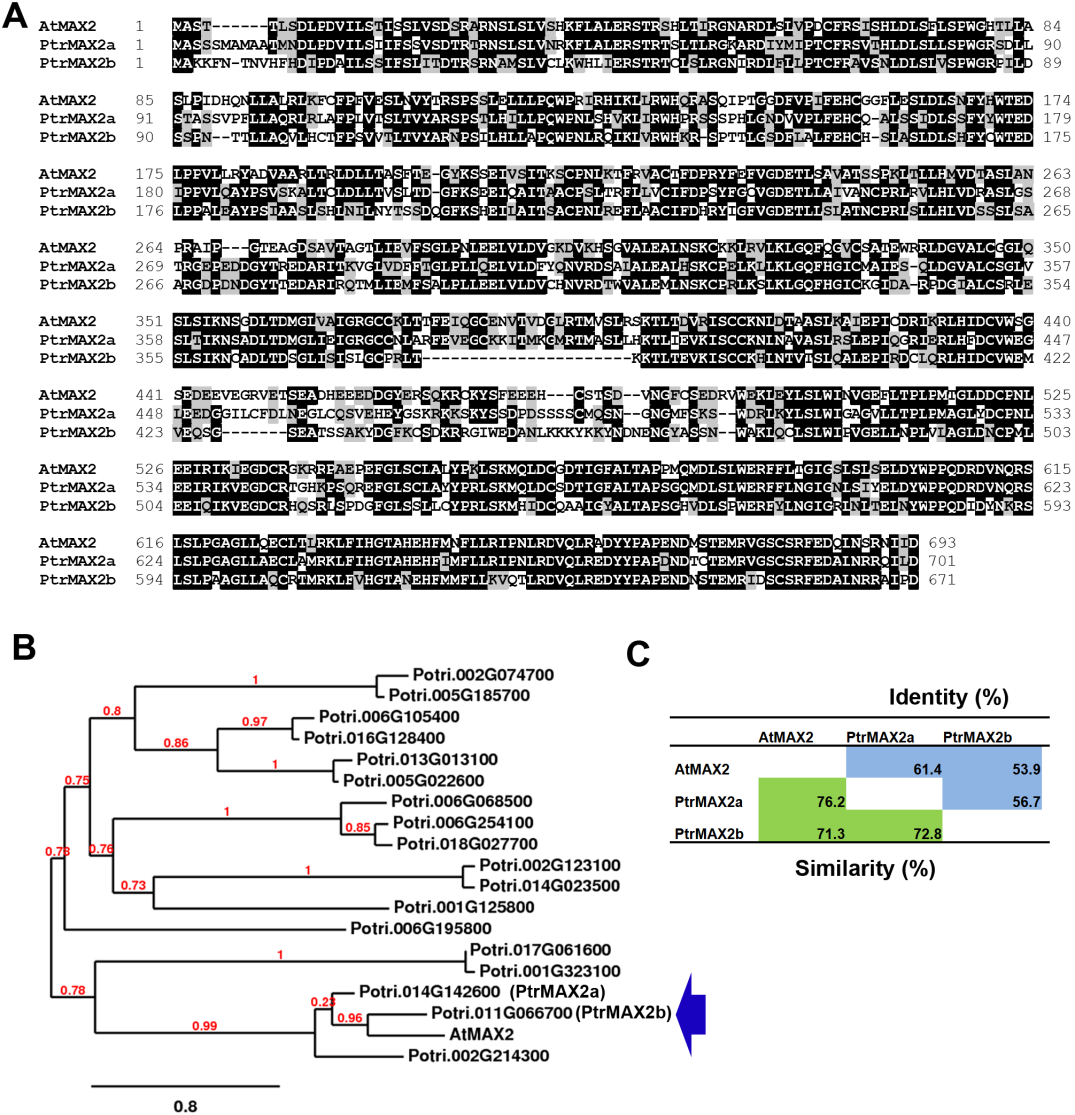
Bioinformatic analysis of MAX2 proteins from *Populus* and *Arabidopsis*. (**A**) Amino acid sequence alignment. (**B**) Phylogenetic analysis of *Populus* sequence homologues of *Arabidopsis* MAX2 protein. (**C**) Amino acid sequence similarity and identity.

The two closest *Populus* sequence homologues of Arabidopsis MAX2 (AtMAX2) were encoded by loci Potri.014G142600 and Potri.011G066700, designated as *PtrMAX2a* and *PtrMAX2b*, respectively (Figure 2). A third homolog, Potri.002G214300 consisting of 316 aa is only half the size of AtMAX2 (693 aa). Thus, it was not included in our further analysis. PtrMAX2a showed approximately 76% similarity and 61% identity with AtMAX2 at the amino acid level. PtrMAX2b showed approximately 71% similarity and 54% identity with AtMAX2 at the amino acid level. PtrMAX2a and PtrMAX2b each other shared approximately 73% similarity and 57% identity at the amino acid level. The number of amino acids of PtrMAX2a (701 aa) and PtrMAX2b (671 aa) are comparable to that of AtMAX2 (693 aa). No other *Populus* proteins showed more than 32% similarity with AtMAX2 at the amino acid level.

**Figure 3:**
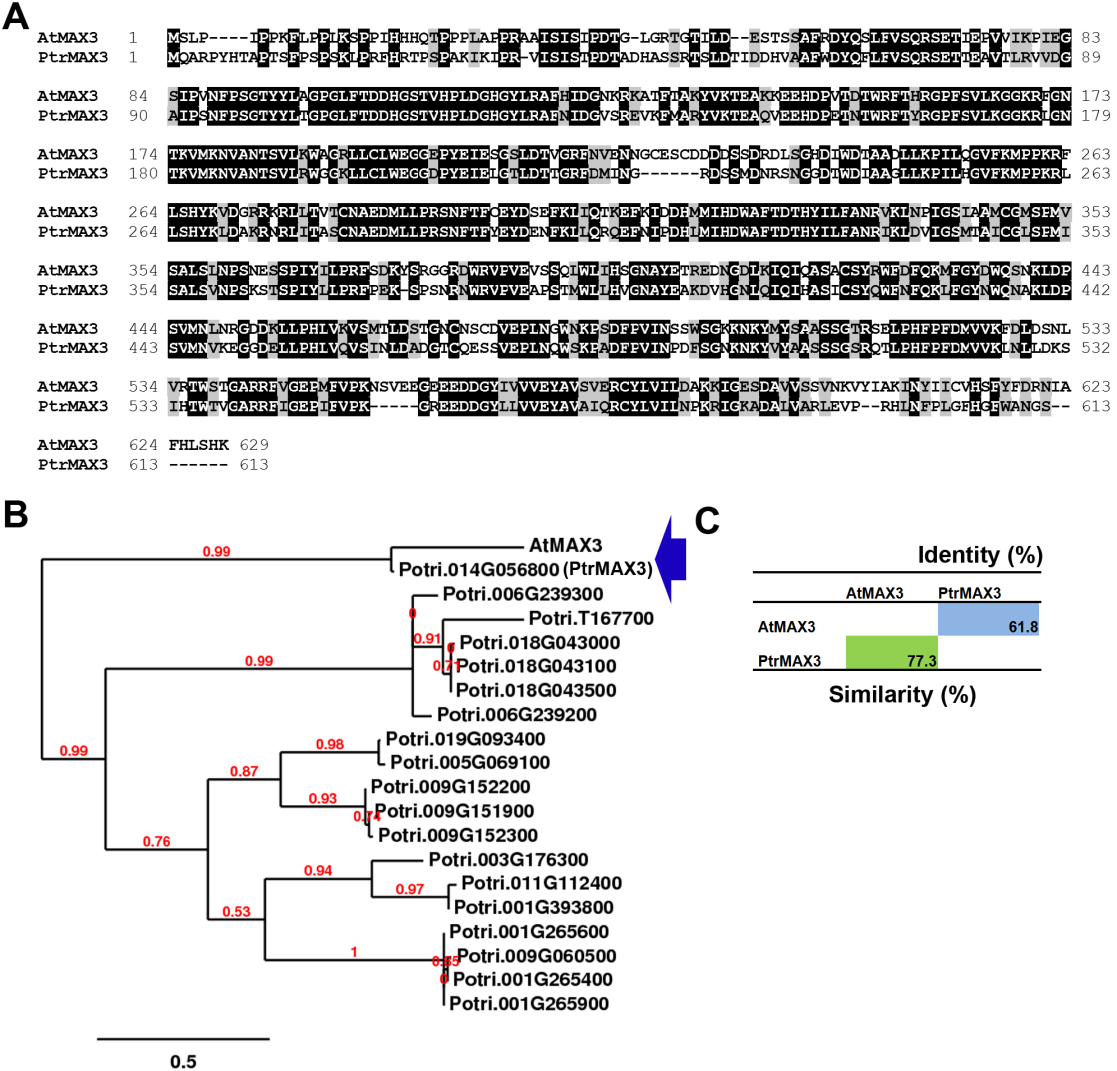
Bioinformatic analysis of MAX3 proteins from *Populus* and *Arabidopsis*. (**A**) Amino acid sequence alignment. (**B**) Phylogenetic analysis of *Populus* sequence homologues of *Arabidopsis* MAX3 protein. (**C**) Amino acid sequence similarity and identity.

Arabidopsis MAX3 (AtMAX3) has only one close sequence homologue in *Populus* encoded by loci Ptri.014G056800, designated as *PtrMAX3* (Figure 3). PtrMAX3 showed approximately 77% similarity and 62% identity with AtMAX3 at the amino acid level. The number of amino acids of PtrMAX3 (613 aa) is comparable to that of AtMAX3 (629 aa). No other *Populus* proteins showed more than 35% similarity to AtMAX3 at the amino acid level.

**Figure 4:**
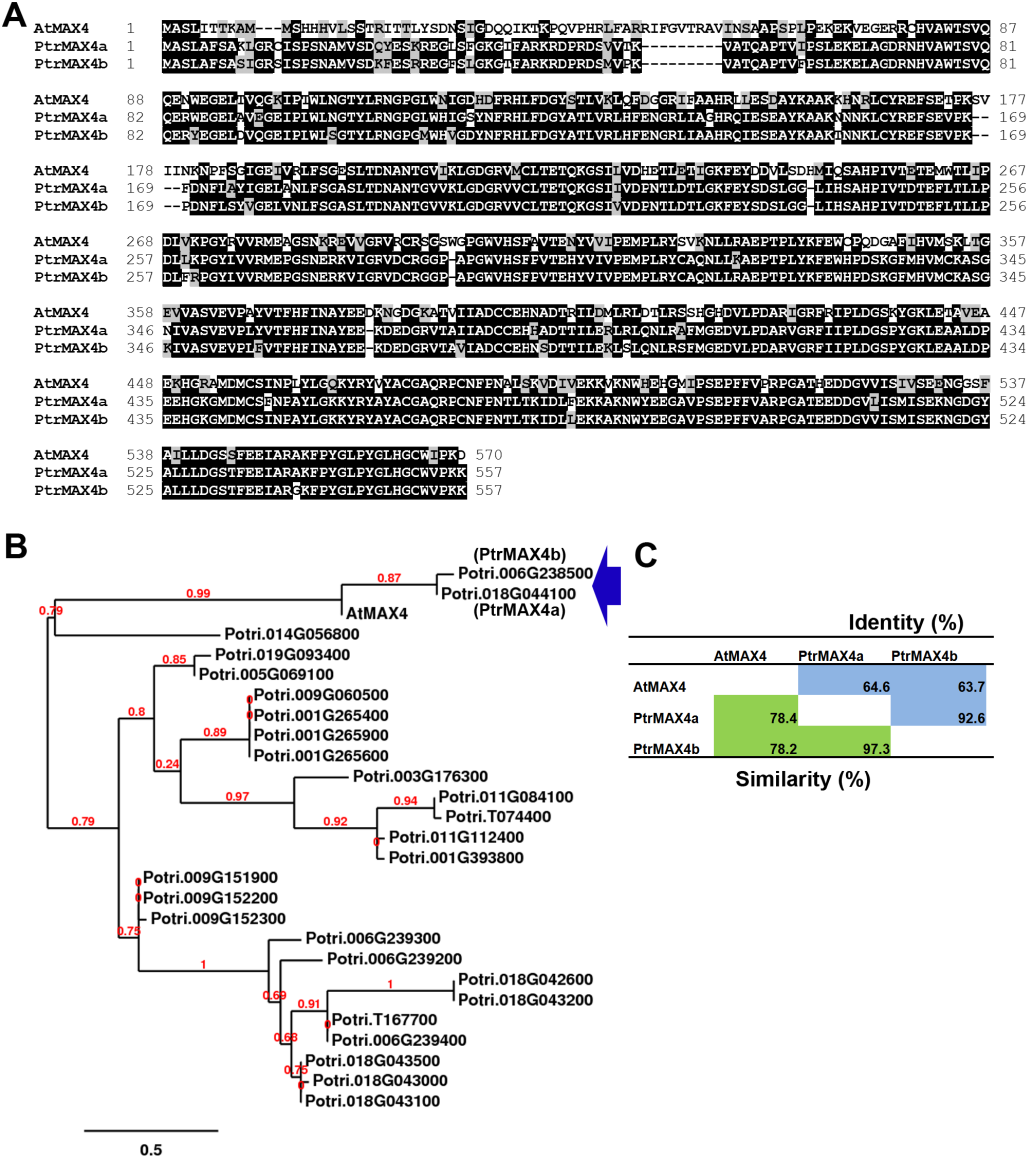
Bioinformatic analysis of MAX4 proteins from *Populus* and *Arabidopsis*. (**A**) Amino acid sequence alignment. (**B**) Phylogenetic analysis of *Populus* sequence homologues of *Arabidopsis* MAX4 protein. (**C**) Amino acid sequence similarity and identity.

Arabidopsis MAX4 (AtMAX4) has two close sequence homologues in *Populus* encoded by loci Potri.018G044100 and Potri.006G238500, designated as *PtrMAX4a* and *PtrMAX4b*, respectively (Figure 4). Both *PtrMAX4a* and *PtrMAX4b* have two transcript variants based on the current annotation by Phytozome. The primary variant (e.g., Potri.018G044100.1 and Potri.006G238500.1) were used for subsequent analysis. PtrMAX4a showed approximately 78% similarity and 65% identity with AtMAX4 at the amino acid level. PtrMAX4b showed approximately 78% similarity and 64% identity with AtMAX4 at the amino acid level. PtrMAX4a and PtrMAX4b each other shared approximately 97% similarity and 93% identity at the amino acid level. The number of amino acids of PtrMAX4a (557 aa) and PtrMAX4b (557 aa) are comparable to that of AtMAX4 (570 aa). No other *Populus* proteins showed more than 42% similarity to AtMAX4 at the amino acid level.

In total, we identified two *Populus* sequence homologues each for Arabidopsis MAX1, MAX2 and MAX4, and one *Populus* sequence homologues for Arabidopsis MAX3.

### Tissue and Organ Expression Patterns of *Populus MAX* Genes

**Figure 5:**
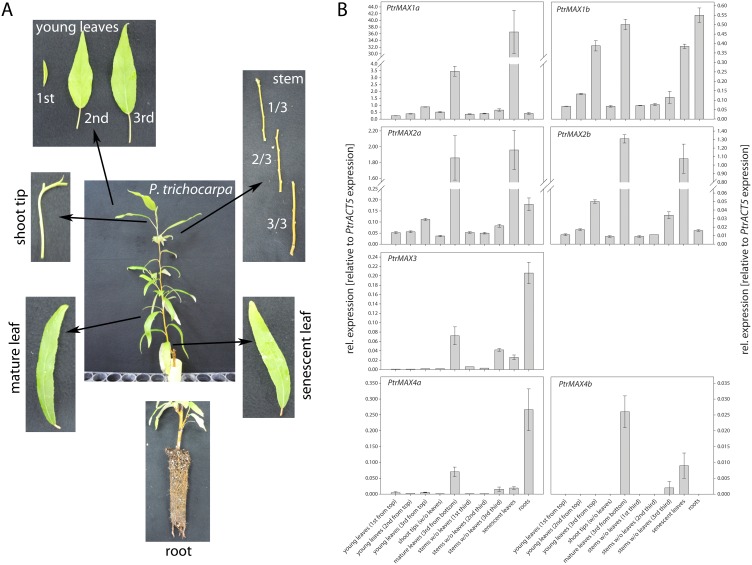
Expression of *Populus MAX* homologous genes across various tissues and organs. (**A**) Illustration of tissues and organs used for expression analysis. (**B**) Quantitative RT-PCR data. Shown are means ± S.E. of three biological replicates.

Subsequently, we examined the expression patterns of each *PtrMAX* gene across various tissues and organs. We used qRT to examine the transcript level of each *PtrMAX* gene and normalized their transcript levels against the transcript level of *PtrACT5*. We made three interesting observations. Firstly, each *PtrMAX* gene was expressed at different levels with *PtrMAX1a* at the highest level and *PtrMAX4b* at the lowest level in most tissues and organs (Figure 5). Secondly, differential expression in terms of transcript level was observed between *PtrMAX* paralogous genes. For example, *PtrMAX1a* was expressed over 10-fold higher than *PtrMAX1b* across most tissues and organs. Similarly, *PtrMAX4a* was expressed over 10-fold higher than *PtrMAX4b* across most tissues and organs. In contrast, *PtrMAX2a* and *PtrMAX2b* were expressed at similar levels in most tissues and organs. Finally, differences in tissue and organ expression patterns were observed between *PtrMAX* paralogous genes. For example, *PtrMAX4a* was highly expressed in roots but the transcript of *PtrMAX4b* was undetectable in roots (Figure 5).

### Genetic Complementation of Arabidopsis *max* Mutants with *Populus MAX* Genes

In order to further test whether *PtrMAX* genes may function similarly as *AtMAX* genes, we conducted genetic complementation studies by heterologously expressing each *PtrMAX* gene in corresponding Arabidopsis *max* mutants. The expression of each *PtrMAX* gene was driven by the constitutive *35S* promoter [55]. At least 20 independent transgenic lines were selected from each transformation and two independent transgenic lines were selected for further analysis. We used the number of primary rosette-leaf branches as a phenotype to determine whether heterologous expression of each *PtrMAX* gene could rescue the corresponding Arabidopsis *max* mutants.

**Figure 6:**
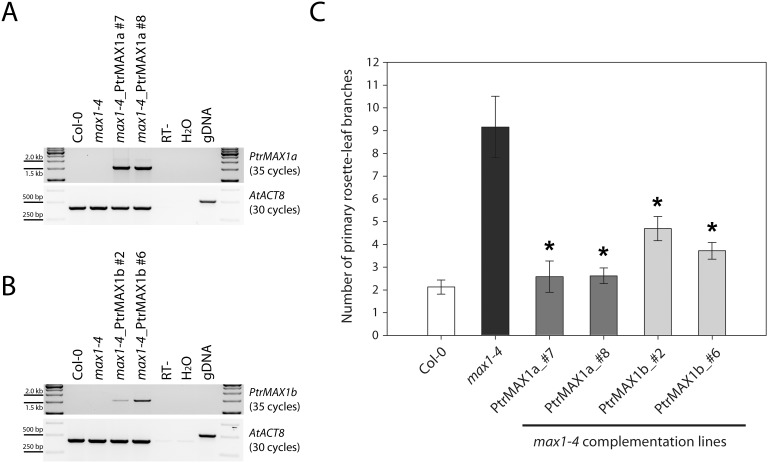
Genetic complementation of *Arabidopsis max1* mutants with *Populus MAX1* genes. (**A**) RT-PCR analysis of *35S:PtrMAX1a* transgenic lines. (**B**) RT-PCR analysis of *35S:PtrMAX1b* transgenic lines. (**C**) Number of primary rosette-leaf branches. Shown are average numbers of primary rosette-leaf branches from at least 10 individual plants ± S.E. *, significant difference from *max1-4*, p<0.05.

For the test of *PtrMAX1* genes, we used a homozygous T-DNA insertional *max1* mutant allele designated as *max1-4* as the transformation background. In this mutant allele, a T-DNA is insertion in the 1^st^ exon, 74 bp downstream of the start codon of MAX1 (Figure S1). RT-PCR analysis indicated that the full-length transcript of *MAX1* was absent in *max1-4* mutant, suggesting that it is likely a loss-of-function allele (Figure S1). Consistent with the RT-PCR result, *max1-4* mutants displayed high number of primary rosette-leaf branches (Figure S1). For the complementation, the full-length ORF of *PtrMAX1a* and *PtrMAX1b* was each cloned into the binary plant expression vector pGWB502Ω (2×CaMV35SΩ) [50] and transformed into *max1-4* mutant background via *Agrobacterium*-mediated transformation. RT-PCR analysis indicated that in the transgenic lines (lines #7 and #8 for *PtrMAX1a*, and lines #2 and #6 for *PtrMAX1b*), *PtrMAX1a* or *PtrMAX1b* transgene was expressed in the Arabidopsis *max1-4* mutant background (Figure 6A, B). We counted the number of primary rosette-leaf branches and compared it with that of Columbia-0 (Col-0) wild type and *max1-4* mutants which were grown side-by-side with transgenic lines under identical conditions. We found that the number of primary rosette-leaf branches in the transgenic lines was largely reverted to a similar number as in the wild type. Under our growth conditions, Col-0 produced about two primary rosette-leaf branches whereas *max1-4* mutants produced about nine primary rosette-leaf branches. The *PtrMAX1a* transgenic lines #7 and #8 produced approximately two primary rosette-leaf branches and *PtrMAX1b* transgenic lines #2 and #6 produced about four primary rosette-leaf branches (Figure 6C). These results indicated that both *PtrMAX1a* and *PtrMAX1b* were able to complement or partially complement the shoot branching phenotypes of Arabidopsis *max1-4* mutant.

**Figure 7:**
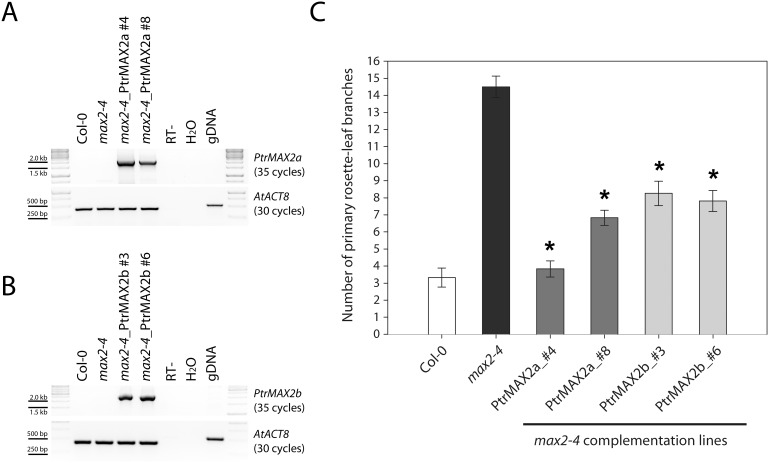
Genetic complementation of *Arabidopsis max2* mutants with *Populus MAX2* genes. (**A**) RT-PCR analysis of *35S:PtrMAX2a* transgenic lines. (**B**) RT-PCR analysis of *35S:PtrMAX2b* transgenic lines. (**C**) Number of primary rosette-leaf branches. Shown are average numbers of primary rosette-leaf branches from at least 10 individual plants ± S.E. *, significant difference from *max2-4*, p<0.05.

For the test of *PtrMAX2* genes, we used a T-DNA insertional *max2* mutant allele, *max2-4*, as the transformation background. The *max2-4* allele had been previously described by Umehara et al. [2] and Ha et al. [56] but the genotyping and branching phenotype were not reported. These data are provided here as supplemental materials (Figure S2). In this mutant allele, a T-DNA insertion site was found within the 1^st^ exon. RT-PCR analysis indicated that the full-length transcript of *MAX2* was absent in *max2-4* mutant, suggesting that it is likely a loss-of-function allele. Consistent with the RT-PCR result, *max2-4* mutants displayed more shoot branches (Figure S2). For the complementation, the full-length ORF of *PtrMAX2a* and *PtrMAX2b* was each cloned into the binary plant expression vector pGWB502Ω and transformed into *max2-4* mutant background. RT-PCR analysis indicated that in the transgenic lines (lines #4 and #8 for *PtrMAX2a*, and lines #3 and #6 for *PtrMAX2b*), *PtrMAX2a* or *PtrMAX2b* transgene was expressed in the Arabidopsis *max2-4* mutant background (Figure 7). Compared with *max2-4* mutants, the number of primary rosette-leaf branches in the transgenic lines was significantly reduced and in line #4 of *PtrMAX2a*, the number was reverted to a similar number in the wild type (Figure 7C). These results indicated that both *PtrMAX2a* and *PtrMAX2b* were able to complement or partially complement the shoot branching phenotypes of Arabidopsis *max2-4* mutant.

**Figure 8:**
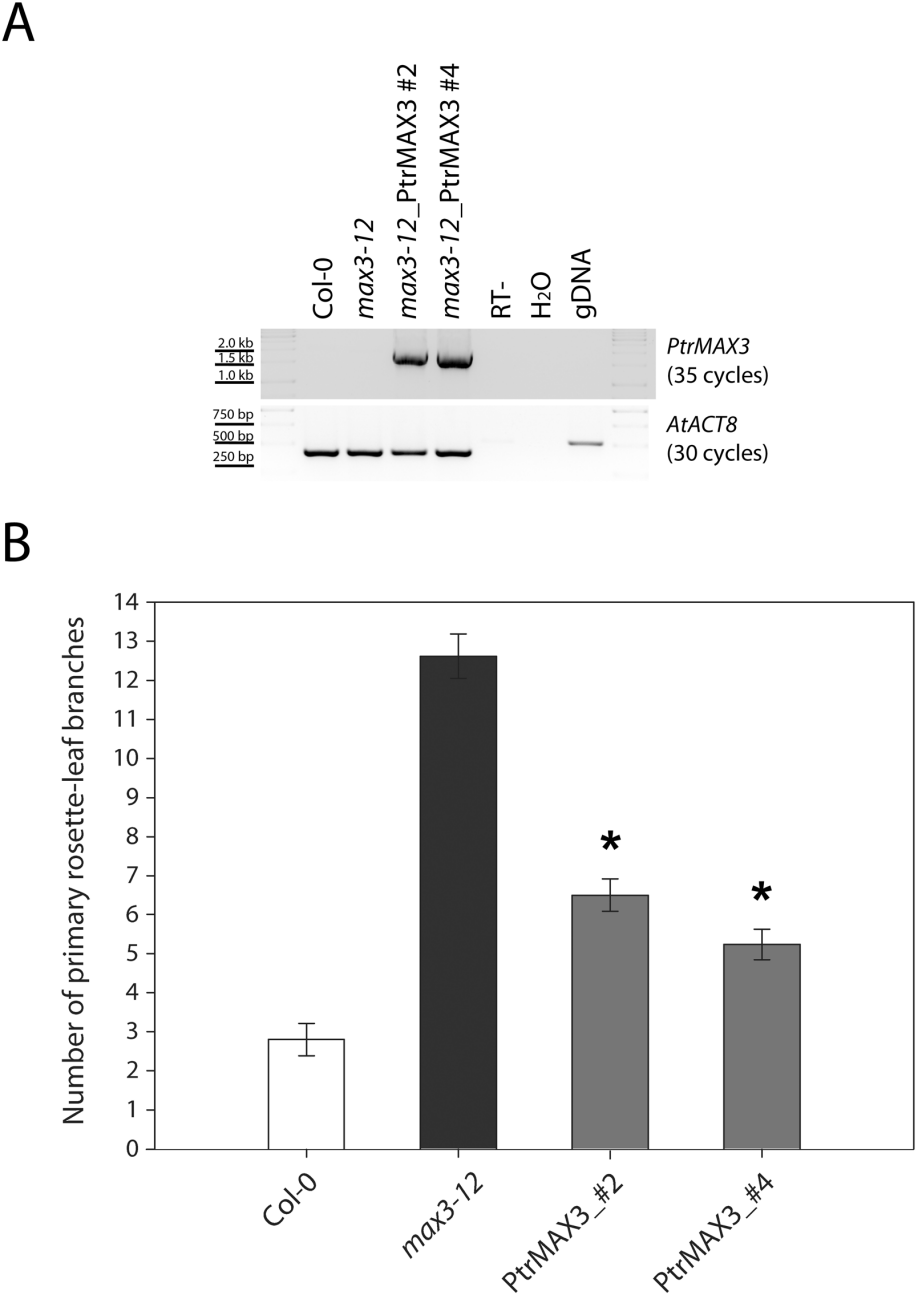
Genetic complementation of *Arabidopsis max3* mutants with *Populus MAX3* gene. (**A**) RT-PCR analysis of *35S:PtrMAX3* transgenic lines. (**B**) Number of primary rosette-leaf branches. Shown are average numbers of primary rosette-leaf branches from at least 10 individual plants ± S.E. *, significant difference from *max3-12*, p<0.05.

For the test of *PtrMAX3* gene, we used a T-DNA insertional *max3* mutant allele, *max3-12*, as the transformation background. *max3-12* was previously described by Umehara et al. [2] and the genotypic and phenotypic data had been reported by Li et al. [47]. The full-length ORF of *PtrMAX3* was cloned into the binary plant expression vector pGWB502Ω and transformed into *max3-12* mutant background. RT-PCR analysis indicated that in the transgenic lines (lines #2 and #4), *PtrMAX3* transgene was expressed in the Arabidopsis *max3-12* mutant background (Figure 8). Compared with *max3-12* mutants, the number of primary rosette-leaf branches in the transgenic lines was significantly reduced but was not completely reverted to wild-type branch number (Figure 8C). These results indicated that *PtrMAX3* was able to partially complement the shoot branching phenotypes of Arabidopsis *max3-12* mutant.

**Figure 9:**
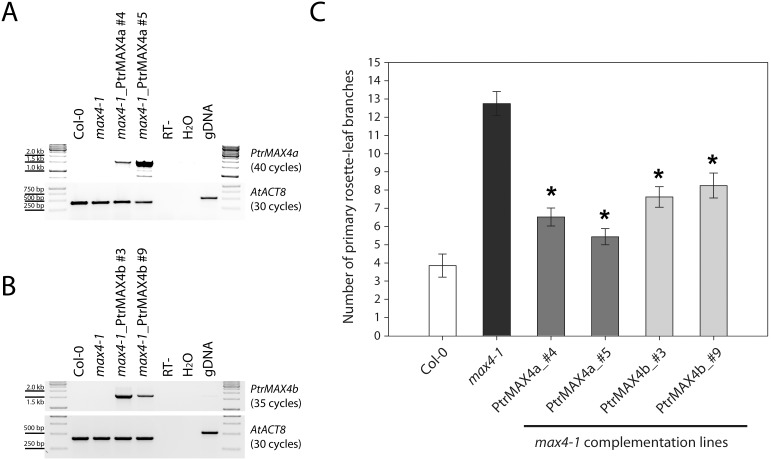
Genetic complementation of *Arabidopsis max4* mutants with *Populus MAX4* genes. (**A**) RT-PCR analysis of *35S:PtrMAX4a* transgenic lines. (**B**) RT-PCR analysis of *35S:PtrMAX4b* transgenic lines. (**C**) Number of primary rosette-leaf branches. Shown are average numbers of primary rosette-leaf branches from at least 10 individual plants ± S.E. *, significant difference from *max4-1*, p<0.05.

For the test of *PtrMAX4* genes, *35S:PtrMAX4a* and *35S:PtrMAX4b* transgenic lines were generated using Arabidopsis *max4-1* mutant [31] as background. RT-PCR analysis indicated that in the *35S:PtrMAX4* transgenic lines, *PtrMAX4a* (lines #4 and #5) or *PtrMAX4b* (lines #3 and #9) transgene was expressed (Figure 9). Compared with *max4-1* mutants, the numbers of primary rosette-leaf branches were significantly reduced in these transgenic lines but were not completely reverted to wild-type branch number (Figure 9C). These results indicated that both *PtrMAX4a* and *PtrMAX4b* were able to partially complement the shoot branching phenotype of Arabidopsis *max4* mutants.

## Discussion

SLs are a new class of plant hormones controlling shoot branching. Due to the importance of shoot branching in the determination of photosynthetic light use efficiency and biomass yield in woody perennial trees, we wanted to examine whether the strigolactone pathway is conserved in the woody model plant *Populus*. We report the identification of sequence homologues of four founding members of SL pathway genes, namely *MAX1*, *MAX2*, *MAX3* and *MAX4*. Furthermore, we provide genetic evidence that these *Populus MAX* genes likely function similarly in controlling shoot branching.

Due to recent genome duplication, in general, each Arabidopsis gene can have two paralogous genes in *Populus*. As such, we found two *Populus* paralogous genes for *MAX1*, *MAX2* and *MAX4* (Figures 1, 2 and 4). The exception here is *MAX3*; only one *MAX3* homologous gene was identified in the *Populus* genome (Figure 3). Our gene expression analysis indicated that *Populus MAX* paralogous genes are differentially expressed across various tissues and organs, in terms of expression level and expression patterns. Over 10-fold differences in transcript level were observed between *PtrMAX1a* and *PtrMAX1b*, and between *PtrMAX4a* and *PtrMAX4b* (Figure 5). It remains unclear whether differences in expression level or pattern of *Populus MAX* genes may result in differences in their functioning. When their expression was driven by the constitutive *35S* promoter, *PtrMAX1* and *PtrMAX4* paralogous genes could rescue the shoot branching phenotype of the corresponding Arabidopsis *max* mutants (Figure 6, Figure 9), suggesting that both paralogous genes could function similarly. The expression analysis also indicated that *PtrMAX3* and *PtrMAX4a* were expressed at the highest level in roots (Figure 5). These results are consistent with the view that roots represent one of the major biosynthesis sites for SLs (reviewed by [25]). This has been demonstrated in a grafting experiment where grafting wild-type Arabidopsis rootstocks to *max1*, *max3*, or *max4* scions were able to restore a wild-type branching pattern to the mutant shoots [30].

For the complementation tests, we found that all *PtrMAX* genes were able to rescue or partially rescue the shoot branching phenotypes of corresponding Arabidopsis *max* mutants (Figures 6, 7, 8 and 9). For *PtrMAX1* and *PtrMAX2*, we could identify transgenic lines in which the numbers of primary rosette-leaf branches were completely reverted to wild-type level (Figure 6, Figure 7), suggesting that *PtrMAX1* and *PtrMAX2* may function equivalently to Arabidopsis *MAX1* and *MAX2*, respectively. On the other hand, although the numbers of primary rosette-leaf branches in *PtrMAX3* and *PtrMAX4* transgenic lines were significantly reduced when compared with Arabidopsis *max3* and *max4* mutants, respectively, the numbers were not completely reverted to wild-type level. There are several possible explanations. Firstly, the expression level of *PtrMAX3* and *PtrMAX4* in the transgenic lines may not be high enough to render complete rescue. Secondly, PtrMAX3 and PtrMAX4 may function similarly, but not equivalently, as Arabidopsis MAX3 and MAX4, respectively. PtrMAX3 showed approximately 77% similarity and 62% identity with MAX3 (Figure 3) and PtrMAX4 showed approximately 78% similarity and 65% identity with AtMAX4 at the amino acid level (Figure 4). It is unclear whether differences in amino acid sequence between *Populus* and *Arabidopsis* MAX3 and MAX4 may have contributed to such partial rescue phenotypes in the transgenic lines. Finally, it is also possible that there may be other MAX3 and MAX4 homologues in *Populus* though no other *Populus* proteins showed more than 35% similarity with AtMAX3 and no other *Populus* proteins showed more than 42% similarity with AtMAX4 at the amino acid level. This deserves further investigation.

In addition to *MAX* genes, several other genes are involved in SL biosynthesis or signaling such as D27 [33], [34] and D14 [36], [38]–[40]. It would be helpful to identify sequence homologs of each of those genes in *Populus* and conduct genetic complementation studies similar to what have been described in this study in order to strengthen the view that strigolactone pathway exists and operates in the woody perennial plant *Populus*. Our preliminary analysis indicated sequence homologues of AtD14 (encoded by locus At3g03990) are present in the genome of *Populus*. For example, two proteins encoded by locus Potri.002G118900 and Potri.014G016500 showed about 89% and 88% similarity with AtD14 at the amino acid level. Our preliminary analysis indicated that sequence homologues of AtD27 (encoded by locus At1g03055) are also present in the genome of *Populus*. For example, a protein encoded by locus Potri.005G216400 showed about 59% similarity with AtD27 at the amino acid level. In order to fully assign SL pathway in *Populus*, future studies should focus on the functional characterization of these SL pathway genes (e.g. generating *Populus* transgenic lines), the determination of SL molecules, biosynthetic intermediates and derivatives, and the determination of the physiological roles of SLs in *Populus*.

In summary, this study provided a preliminary characterization of strigolactone pathway genes in the model woody plant *Populus*. Sequence homologues of those four founding members of Arabidopsis SL pathway genes have been identified in *Populus*. Gene expression analysis indicated that *Populus MAX* paralogous genes are differentially expressed across various tissues and organs. Genetic complementation studies indicated *Populus MAX* genes are able to complement or partially complement the shoot branching phenotypes of corresponding Arabidopsis *max* mutants. These findings lay a foundation for further characterization of SL pathway and its functions in woody perennial plants.

## Supporting Information

Figure S1 **Arabidopsis** ***max1-4*** **mutant.** (**A**) T-DNA insertion site. (**B**) PCR genotyping. (**C**) RT-PCR analysis. (**D**) Shoot branching phenotypes. (**E**) Number of primary rosette-leaf branches. Shown are average numbers of primary rosette-leaf branches from at least 10 individual plants ± S.E. *, significant difference from Col-0, p<0.05.
(TIF)

Figure S2 **Arabidopsis** ***max2-4*** **mutant.** (**A**) T-DNA insertion site. (**B**) PCR genotyping. (**C**) RT-PCR analysis. (**D**) Shoot branching phenotypes. (**E**) Number of primary rosette-leaf branches. Shown are average numbers of primary rosette-leaf branches from at least 10 individual plants ± S.E. *, significant difference from Col-0, p<0.05.
(TIF)

Table S1 **A list of primers used in this study.**
(DOC)
