# Supplementary figures and images for: Characterization of MORE AXILLARY GROWTH Genes in Populus
Source: PLoS One. 2014 Jul 18;9(7):e102757. doi: 10.1371/journal.pone.0102757 (PMC4103879; doi:10.1371/journal.pone.0102757)

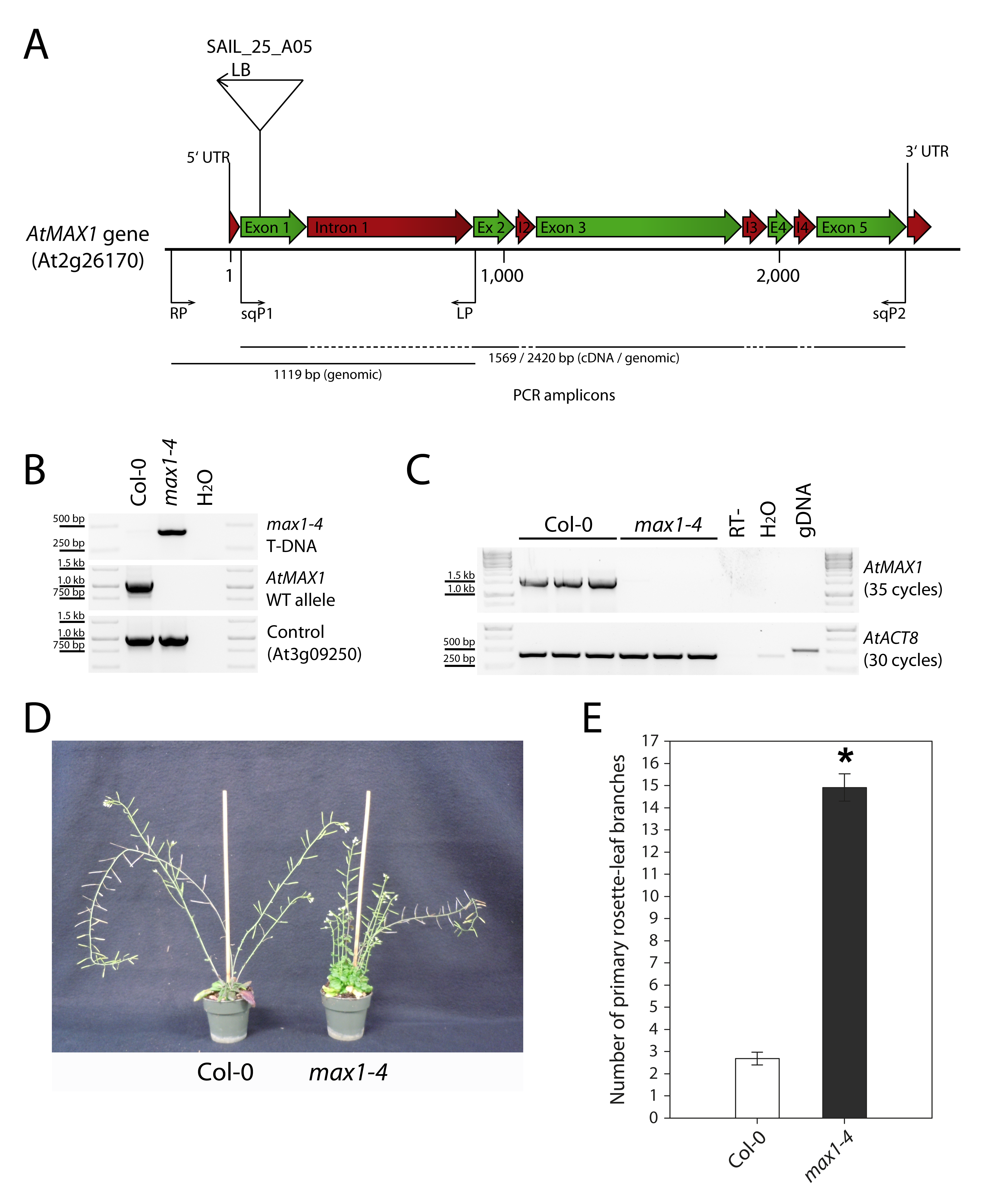

Supplement: Figure S1 — Arabidopsis max1-4 mutant. (A) T-DNA insertion site. (B) PCR genotyping. (C) RT-PCR analysis. (D) Shoot branching phenotypes. (E) Number of primary rosette-leaf branches. Shown are average numbers of primary rosette-leaf branches from at least 10 individual plants ± S.E. *, significant difference from Col-0, p<0.05. (TIF) [file pone.0102757.s001.tif]

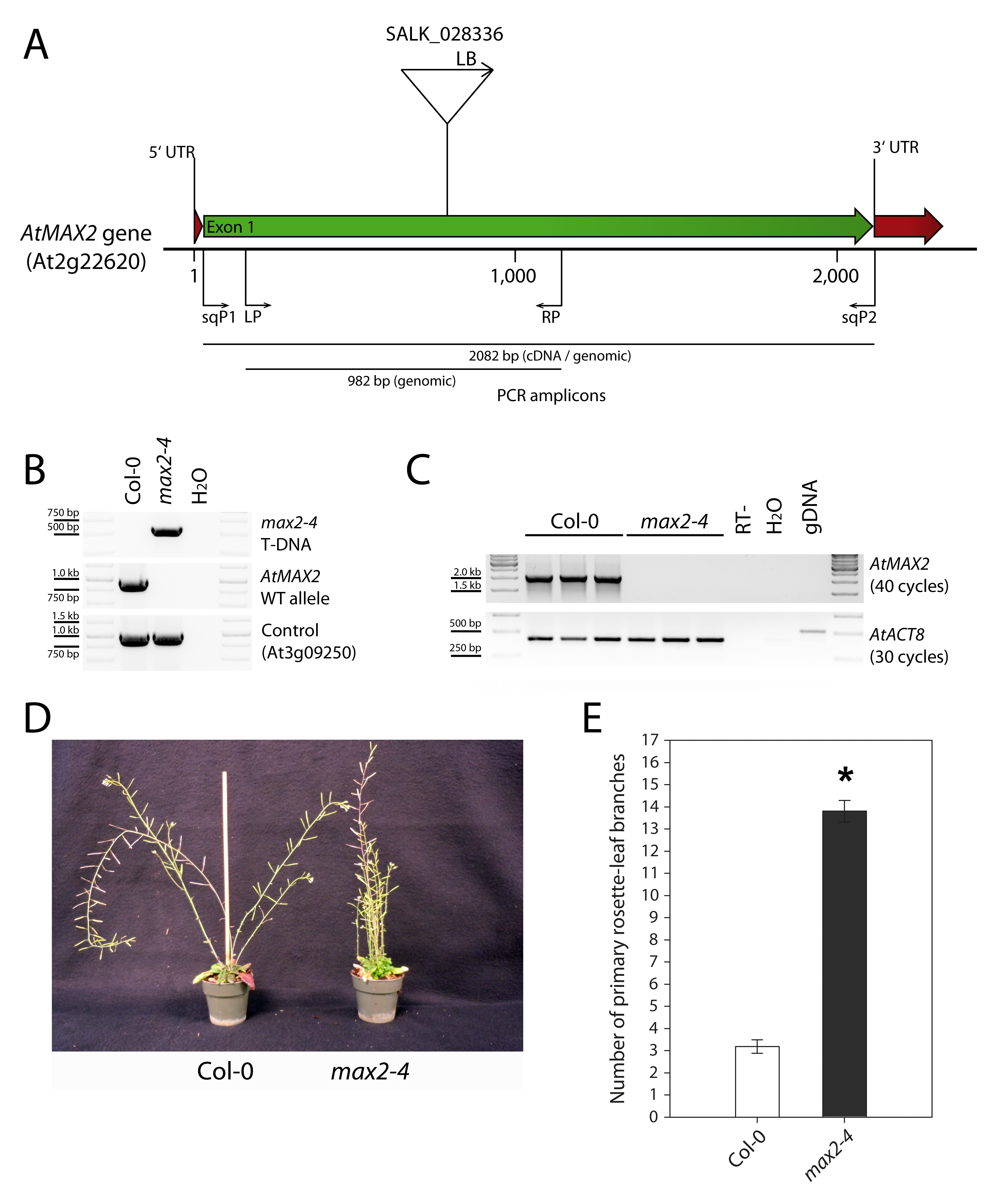

Supplement: Figure S2 — Arabidopsis max2-4 mutant. (A) T-DNA insertion site. (B) PCR genotyping. (C) RT-PCR analysis. (D) Shoot branching phenotypes. (E) Number of primary rosette-leaf branches. Shown are average numbers of primary rosette-leaf branches from at least 10 individual plants ± S.E. *, significant difference from Col-0, p<0.05. (TIF) [file pone.0102757.s002.tif]
