# Supplementary material for: Characterization of MORE AXILLARY GROWTH Genes in Populus
Source: PLoS One. 2014 Jul 18;9(7):e102757. doi: 10.1371/journal.pone.0102757 (PMC4103879; doi:10.1371/journal.pone.0102757)
Supplement: Table S1 — A list of primers used in this study. (DOC) [file pone.0102757.s003.doc]

**Table S1.** A list of primers used in this study

|  |  |
| --- | --- |
| **Primers** | **5’-3’ Sequences** |
|  |  |
| **Primers for cloning** |  |
|  |  |
| PtrMAX1a | CACCAAAATGGATTTACAGGTCTTGTTT;  TCAAGTTCGTTTTATGATTCT |
| PtrMAX1b | CACCAAAATGAGTACGGATTTACAGGTA;  TCAAGTTCGTTTTACAATTCT |
| PtrMAX2a | CACCAAAATGGCTGCTACCATGAACGAT;  TCAGTCGAGGATCTGACGCCT |
| PtrMAX2b | CACCAAAATGGCTAAAAAATTTAACACT;  TCAATCAGGAATCGCACGCCT |
| PtrMAX3 | CACCAAAATGCAAGCAAGACCATACCAT;  TTAAGAACCGTTGGCCCAAAAACC |
| PtrMAX4a | CACCAAAATGGTTTCTGATCAGTATGAG;  TTACTTCTTCGGCACCCAGCA |
| PtrMAX4b | CACCAAAATGGCTTCCTTGGCATTTTCC;  TTATTTCTTTGGCACCCAGCA |
|  |  |
| **Primers for qRT-PCR** |  |
|  |  |
| PtrMAX1a | GGATCTGGTTAGCACCTGGA;  GGTCCGATTCCAAAGGGTAT |
| PtrMAX1b | GGATCTGGTTAGCACCTGGA;  TCTCCGGCTTGAACTTGTCT |
| PtrMAX2a | GTCGAGGGAGACTGCAGAAC;  AGCAAGGCAGCTTAATCCAA |
| PtrMAX2b | TTAATTCCAAGTGCCCAAGG;  CCGTGAAACTGTCCCAACTT |
| PtrMAX3 | TCCATGACTGGGCATTTACA;  ATCAAGCTTAATGCGGTTGG |
| PtrMAX4a | TGCTTATGCTTGTGGAGCAC;  TAGTGAGGGTGTTGGGGAAG |
| PtrMAX4b | GTGATGGACGTGTTGTTTGC;  TTTCCCTAACGTGTCCAAGG |
| PtrACT5 | GCATCCACGAGACCACATAC;  ATAGAGCCACCGATCCAGAC |
|  |  |
| **Primers for sqRT-PCR** |  |
|  |  |
| PtrMAX1a | Same as Cloning Primers |
| PtrMAX1b | Same as Cloning Primers |
| PtrMAX2a | Same as Cloning Primers |
| PtrMAX2b | Same as Cloning Primers |
| PtrMAX3 | Same as Cloning Primers |
| PtrMAX4a | Same as Cloning Primers |
| PtrMAX4b | Same as Cloning Primers |
| AtACT8 | ATGAAGATTAAGGTCGTGGCA;  TCCGAGTTTGAAGAGGCTAC |
|  |  |
| **Primers for Arabidopsis *max* mutant genotyping and sequencing** | |
| *max1-4* |  |
|  |  |
| SAIL_LB | TAGCATCTGAATTTCATAACCAATCTCGATACAC |
| RP | AATTAATACGGATTCCGTGCG |
| LP | TCCCCATCTGAAATCTGTTTG |
| sqP1 | ATGAAGACGCAACATCAATGGT |
| sqP2 | TCAGAATCTTTTGATGGTTCTGAG |
| Control (At3g09250) | AGAGCCCATTGATATTGGATG;  ACAGGAGAAGCATGGTGAATG |
|  |  |
| *max2-4* |  |
|  |  |
| SALK_LB1.3 | ATTTTGCCGATTTCGGAAC |
| RP | CATCCTCTCCCTATAGCCACC |
| LP | GCTCTCACCTCACTATCCGTG |
| sqP1 | ATGGCTTCCACTACTCTCTC |
| sqP2 | TCAGTCAATGATGTTGCGGC |
|  |  |
|  |  |

Note: Underlined sequences are nucleotide bases introduced for site directed cloning into pENTR™/D-TOPO® (5’-CACC-3’) and consistent Kozak consensus sequences for all transcripts (5’-AAA-3’; Joshi et al. 1997: Context sequences of translation initiation codon in plants. *Plant Mol. Biol.* 35(6), 993-1001).
